# TuberOus SClerosis registry to increase disease Awareness (TOSCA) – baseline data on 2093 patients

**DOI:** 10.1186/s13023-016-0553-5

**Published:** 2017-01-05

**Authors:** John C. Kingswood, Guillaume B. d’Augères, Elena Belousova, José C. Ferreira, Tom Carter, Ramon Castellana, Vincent Cottin, Paolo Curatolo, Maria Dahlin, Petrus J. de Vries, Martha Feucht, Carla Fladrowski, Gabriella Gislimberti, Christoph Hertzberg, Sergiusz Jozwiak, John A. Lawson, Alfons Macaya, Rima Nabbout, Finbar O’Callaghan, Mirjana P. Benedik, Jiong Qin, Ruben Marques, Valentin Sander, Matthias Sauter, Yukitoshi Takahashi, Renaud Touraine, Sotiris Youroukos, Bernard Zonnenberg, Anna C. Jansen, on behalf of TOSCA consortium and TOSCA investigators

**Affiliations:** 1 Sussex Kidney Unit, Royal Sussex County Hospital, Eastern Road, Brighton, BN2 5BE UK; 2 Association Sclérose Tubéreuse de Bourneville, Gradignan, France; 3 Moscow Institute of Pediatrics and Pediatric Surgery, Moscow, Russian Federation; 4 Centro Hospitalar Lisboa Ocidental, Lisbon, Portugal; 5 TSA Tuberous Sclerosis Association, Nottingham, UK; 6 Novartis Farmacéutica SA, Gran Vía Corts Catalanes, Barcelona, Spain; 7 Hôpital Louis Pradel, Claude Bernard University Lyon 1, Lyon, France; 8 Tor Vergata University Hospital, Rome, Italy; 9 Karolinska University Hospital, Stockholm, Sweden; 10 Division of Child and Adolescent Psychiatry, University of Cape Town, Cape Town, South Africa; 11 Universitätsklinik für Kinder-und Jugendheilkunde, Vienna, Austria; 12 Associazione Sclerosi Tuberosa ONLUS, Milan, Italy; 13 European Tuberous Sclerosis Complex Association, In den Birken, Dattein, Germany; 14 Novartis Farma S.p.A, Origgio, Italy; 15 Vivantes-Klinikum Neukölln, Berlin, Germany; 16 Department of Child Neurology, Warsaw Medical University, Warsaw, Poland; 17 The Tuberous Sclerosis Multidisciplinary Management Clinic, Sydney Children’s Hospital, Randwick, NSW Australia; 18 Hospital Universitari Vall d’Hebron, Barcelona, Spain; 19 Department of pediatric neurology, Necker Enfants Malades Hospital, Paris Descartes University, Paris, France; 20 Institute of Child Health, University College London, London, UK; 21 SPS Pediatrična Klinika, Ljubljana, Slovenia; 22 Department of Pediatrics, Peking University People’s Hospital (PKUPH), Beijing, China; 23 Tallinn Children Hospital, Tallinn, Estonia; 24 Klinikverbund Kempten-Oberallgäu gGmbH, Kempten, Germany; 25 National Epilepsy Center, Shizuoka Institute of Epilepsy and Neurological Disorders, NHO, 886 Urushiyama Aoi-ku, Shizuoka, Japan; 26 Hôpital Nord, Saint Etienne, France; 27 “St. Sophia” Children’s Hospital, Athens, Greece; 28 University Medical Center, Utrecht, Netherlands; 29 UZ Brussel VUB, Brussels, Belgium

**Keywords:** Tuberous sclerosis, Registry, Epilepsy, Subependymal giant cell astrocytoma, Angiomyolipoma, TOSCA

## Abstract

**Background:**

Tuberous sclerosis complex (TSC) is a rare autosomal dominant genetic disorder. Many gaps remain in the understanding of TSC because of the complexity in clinical presentation. The **T**uber**O**us **SC**lerosis registry to increase disease **A**wareness (TOSCA) is an international disease registry designed to address knowledge gaps in the natural history and management of TSC. Here, we present the baseline data of TOSCA cohort.

**Methods:**

Patients of any age diagnosed with TSC, having a documented visit for TSC within the preceding 12 months, or newly diagnosed individuals were included. The registry includes a “core” section designed to record detailed background information on each patient including disease manifestations, interventions, and outcomes collected at baseline and updated annually. “Subsections” of the registry recorded additional data related to specific features of TSC.

**Results:**

Baseline “core” data from 2093 patients enrolled from 170 sites across 31 countries were available at the cut-off date September 30, 2014. Median age of patients at enrollment was 13 years (range, 0–71) and at diagnosis of TSC was 1 year (range, 0–69). The occurrence rates of major manifestations of TSC included – cortical tubers (82.2%), subependymal nodules (78.2%), subependymal giant cell astrocytomas (24.4%), renal angiomyolipomas (47.2%), lymphangioleiomyomatosis (6.9%), cardiac rhabdomyomas (34.3%), facial angiofibromas (57.3%), forehead plaque (14.1%), ≥ 3 hypomelanotic macules (66.8%), and shagreen patches (27.4%). Epilepsy was reported in 1748 (83.5%) patients, of which 1372 were diagnosed at ≤ 2 years (78%). Intellectual disability was identified in 451 (54.9%) patients of those assessed. TSC-associated neuropsychiatric disorders (TAND) were diagnosed late, and not evaluated in 30–50% of patients.

**Conclusion:**

TOSCA is the largest clinical case series of TSC to date. It provided a detailed description of the disease trajectory with increased awareness of various TSC manifestations. The rates of different features of TSC reported here reflect the age range and referral patterns of clinics contributing patients to the cohort. Documentation of TAND and LAM was poor. A widespread adoption of the international TSC assessment and treatment guidelines, including use of the TAND Checklist, could improve surveillance. The registry provides valuable insights into the necessity for monitoring, timing, and indications for the treatment of TSC.

**Electronic supplementary material:**

The online version of this article (doi:10.1186/s13023-016-0553-5) contains supplementary material, which is available to authorized users.

## Background

Tuberous sclerosis complex (TSC) is a rare genetic disorder characterized by the development of benign tumors in several organs of the body [1]. The birth incidence of the disorder is approximately 1 in 5800 individuals [2]. TSC is caused by genetic mutations in either of the *TSC1* or *TSC2* genes [3]. Based on routine diagnostic techniques, a pathogenic mutation is detected in up to 85–90% of individuals with a clinical diagnosis of TSC [1]. In the remaining 10–15% patients with ‘no mutation identified’, next generation DNA sequencing (NGS), a high-throughput sequencing, identified mosaic or intronic mutations in *TSC1* or *TSC2* genes in a vast majority suggesting that it is unlikely that a third *TSC* gene exists [4]. Mutations of *TSC1* or *TSC2* gene result in overactivation of the mammalian target of rapamycin (mTOR) complex 1, a key intracellular regulator of cell growth and proliferation, resulting in the hamartomatous lesions found in multiple organs [5, 6]. Recent research has helped us understand the pathophysiology of TSC, which has led to the use of mTOR inhibitors for the treatment of certain manifestations of TSC including subependymal giant cell astrocytomas (SEGAs) and renal angiomyolipomas [7–10]. The recently revised guidelines for the surveillance and management of TSC provided updated recommendations for standard, optimal care for patients [10].

There is, however, still a lack of clarity with respect to the natural history of many of the TSC manifestations, their variability, the age-related expression pattern, and their prognostic roles. Gaps also exist in understanding the rare symptoms and comorbidities of TSC, the relationship between genotype and phenotype, and the various interventions, treatments, and their outcomes. An improved understanding of the natural history of TSC is essential in order to evaluate the benefit-risk ratio of any intervention accurately. **T**uber**O**us **SC**lerosis registry to increase disease **A**wareness (TOSCA) was established as a multicenter, international disease registry with the specific aim to gather clinical data on this rare disease in a systematic and longitudinal way. TOSCA consists of a “core” dataset representing the diagnostic characteristics and core associated clinical features, and “petal projects” which represent detailed sub-projects focusing on specific TSC manifestations. The results from the baseline core data of the TOSCA cohort are reported here.

## Methods

The study methods have been described in detail previously [11]. All patients gave informed consent. TOSCA is a multicenter, international disease registry that was designed to collect data on patients with TSC from many countries worldwide. Patients of any age with a diagnosis of TSC (definite, probable, or possible) and with a documented clinical visit for TSC within the past 12 months or newly diagnosed with TSC were eligible. The certainty of diagnosis was based on the 1998 revised Gomez criteria.[12] The registry consists of a “core” section and subsections or “petals”. The “core” section collected general information on patients’ background which included demographic data, family history, prenatal history, and disease features such as neurological and neuropsychiatric, renal, cardiovascular, pulmonary, dermatological, and others. This information was collected at baseline and is being updated annually. Subsections (“petals”) are being developed as research projects to record in-depth data related to specific disease manifestations. Pediatric and adult sites with specialists in managing one or more aspects of TSC were included in the registry. Patients will be followed up for up to five years and an interim analysis will be conducted every year.

## Results

### Overall findings

**Fig. 1:**
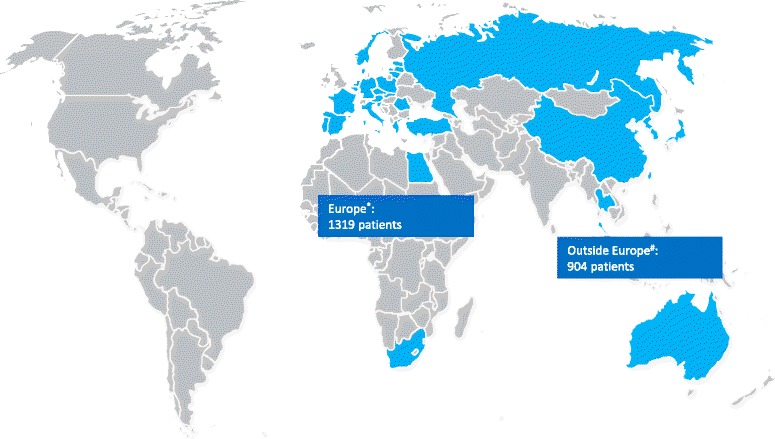
Patients enrolled from different countries in TOSCA (*N* = 2223). ^*^European countries include: Austria, Belgium, Czech Republic, Denmark, Estonia, France, Germany, Greece, Italy, Latvia, Lithunia, Netherlands, Norway, Poland, Portugal, Romonia, Slovakia, Slovenia, Spain, and Sweden. ^#^Outside Europe include: Australia, Israel, Japan, Korea, Russia, South Africa, Mainland China, Hongkong, Macau, Taiwan, Thailand, and Turkey

**Fig. 2:**
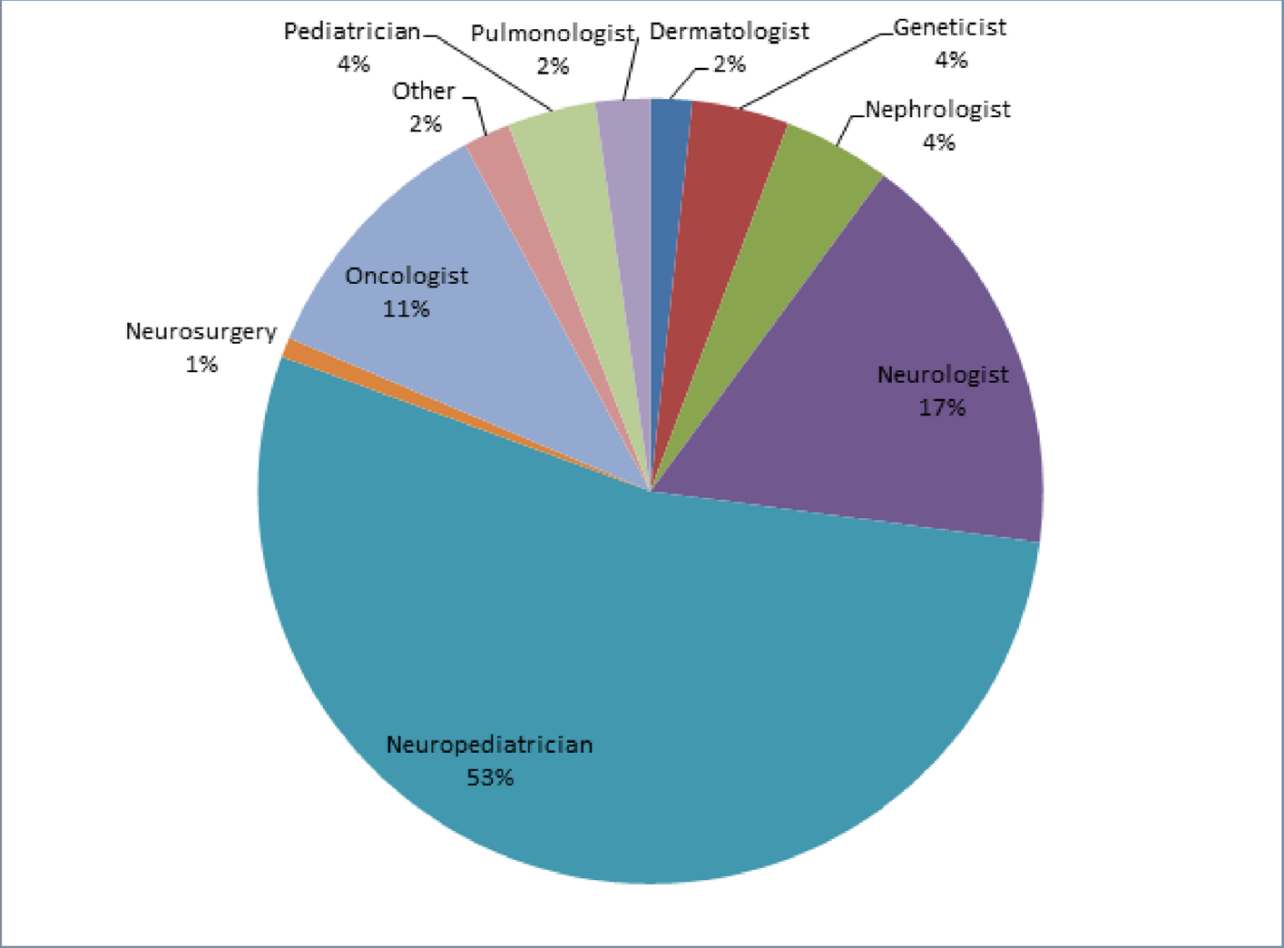
Distribution of TOSCA participants among different specialties (*N* = 2223)

**Fig. 3:**
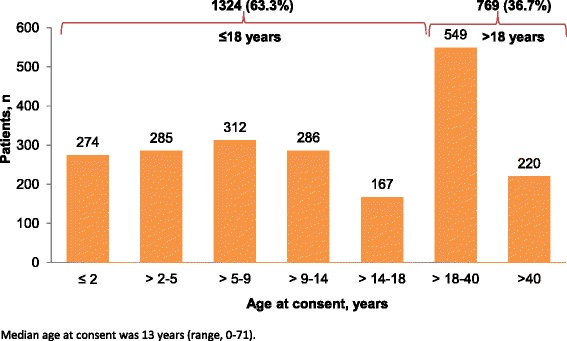
Patient distribution by age for inclusion in TOSCA (*N* = 2093). Median age at consent was 13 years (range 0–71)

TOSCA recruited a total of 2223 patients from 170 centers in 31 countries (Fig. 1), over half of them (57%) from neuropediatric/pediatric clinics (Fig. 2). At the cut-off date September 30, 2014, complete baseline data from 2093 patients (1009 male and 1084 female) were available. Median age at inclusion in TOSCA was 13 years (range, 0–71). Patient distribution by age at inclusion in TOSCA is shown in Fig. 3 (≤18 years, 63.3%; > 18 years, 36.7%). Median age at diagnosis of TSC was 1 year (range 0–69). TSC was diagnosed prenatally in 124 (5.9%) patients. Molecular testing had been performed in 902 (43.1%) patients. *TSC1* mutations were identified in 19.7% of the patients and *TSC2* in 63.3% (Table 1). Only known pathogenic mutations as defined in the Leiden Open Variation Database (LOVD) were counted.[13] Five patients had both *TSC1* and *TSC2* mutations.

**Table 1:** Baseline patient demographics and clinical characteristics (*N* = 2093)

Characteristics	Baseline data
Age at diagnosis of TSC,^a^ years, median (range)	1.0 (0–69)
Gender, n (%)	
Male	1009 (48.2)
Female	1084 (51.8)
Patients with molecular testing, n (%)	902 (43.1)
Genetic testing^b^, n (%^c^)	885 (98.1)
No mutation identified	125 (13.9)
*TSC1* mutation	178 (19.7)
*TSC2* mutation	571 (63.3)
Variation type, n (%)^d^	
Pathogenic mutation	633 (93.8)
Variant of unknown significance	61 (9.0)
Time from TSC clinical diagnosis to molecular testing, months	
Mean (SD)	79.6 (116.78)
Median (range)	22 (0–721)
Patients with prenatal diagnosis, n (%)	124 (5.9)
Biological mother/father evaluated for TSC, n	
Mother	865
Father	753
TSC inherited from one parent, n	
Total	290
Mother	168 (95 clinically)
Father	122 (56 clinically)
Patients with affected relatives, n (%)	
Total	478 (22.8)
1	259 (12.4)
2	116 (5.5)
3	54 (2.6)
> 3	52 (2.5)
Patients with at least one blood relative participating in TOSCA, n (%)	207 (9.9)

*SD* standard deviation, *TSC* tuberous sclerosis complex, *TOSCA* TuberOus SClerosis registry to increase disease Awareness

^a^Data available for 2054 patients; ^b^Information on the type of mutation was missing for 6 patients; 5 patients had both *TSC1* and *TSC2* mutations; ^c^Percentages calculated considering the number of patients with molecular testing as the denominator value. ^d^The count (n) includes 19 patients who had both variation types

### Neurological manifestations

Cortical tubers and subependymal nodules were the most commonly reported neurological manifestations (reported in 82.2 and 78.2% of patients, respectively). A total of 510 (24.4%) patients had ever been diagnosed with SEGA (Table 2). For TOSCA, a broad definition of SEGA was adopted, based on the presence of a lesion near the foramen of Monro without specific criteria for size or growth. Median age at SEGA diagnosis was 8 years (range 0–51) (Additional file 1). SEGA was diagnosed before age 2 years in 132 (26.4%), before age 18 years in 278 (55.6%), and after age 18 years in 90 (18%) patients. SEGA was present at the time of recruitment in TOSCA in 422 (82.7%) patients. Of these, 195 (46.2%) were bilateral and 155 (36.7%) showed growth since the previous scan. Growth was reported in 10 out of 93 patients with SEGA diagnosed after age 18 years. The median time between scans was 1 year (range 0–22).

**Table 2:** Baseline manifestations of TSC reported in TOSCA

Manifestations of TSC	Patients at baseline, n (%)
Neurological	
SEGA	510 (24.4)
Cortical tuber	1721 (82.2)
SEN	1636 (78.2)
Cerebral white matter radial migration lines	429 (20.5)
Renal	
Renal angiomyolipoma	987 (47.2)
Multiple renal cysts	477 (22.8)
Polycystic kidneys	73 (3.5)
Impaired renal function	43 (2.1)
Renal malignancy	24 (1.1)
Pulmonary	
Lymphangioleiomyomatosis	144 (6.9)
Cardiavascular	
Cardiac rhabdomyoma	717 (34.3)
Dermatologic	
≥ 3 hypomelanotic macules	1399 (66.8)
Facial angiofibroma	1199 (57.3)
Shagreen patch	573 (27.4)
Ungual or periungual fibromas	350 (16.7)
Forehead plaque	295 (14.1)
Confetti lesions	179 (8.6)
Ophthalmologic	
Retinal hamartoma	294 (14.0)
Epilepsy	1748 (83.5)

*SEGA* subependymal giant cell astrocytoma, *SEN* subependymal nodule, *TOSCA* TuberOus SClerosis registry to increase disease Awareness

In 207 out of the 510 patients with SEGA, a pathogenic mutation was detected in *TSC1* in 22 patients and *TSC2* in 185 patients. SEGA were bilateral in 18% and growing in 4.5% of patients with mutations in *TSC1* versus in 34 and 16% of patients with mutations in *TSC2*. The majority (70.9%) of the patients with SEGA were asymptomatic at the time of assessment. In symptomatic patients, the most common symptoms/signs were increase in seizure frequency (65 [15.4%]), behavioral disturbances (50 [11.8%]), headache (34 [8.1%]), and regression or loss of cognitive skills (31 [7.3]). A total of 189 (9%) patients received treatment before baseline visit. Median time from SEGA diagnosis to treatment was less than 1 year (range 0–15). The most common treatment modality was surgery (114 [60.3%]), mTOR inhibitor (88 [46.6%]), and ventriculoperitoneal shunt (21 [11.1%]), provided as monotherapy or in combination with other treatments.

Epilepsy was reported in 1748 (83.5%) patients (Table 2). The most common seizure type was focal seizures (1169 [66.9%]). The median age at diagnosis of focal seizures was 1 year. Most of the patients (73%) were diagnosed at or before the age of 2 years. Of the 1144 patients who received treatment, 745 (65.1%) were treated with gamma-aminobutyric acid (GABAergics [as a single agent or in combination with another treatment modality]). Additional treatment modalities as single agents or in combination with other treatment modalities included mTOR inhibitor (80 [7.0%]), surgery (80 [7.0%]), ketogenic diet (49 [4.3%]), vagal nerve stimulation (45 [3.9%]), fructose derivatives (33 [2.9%]), and adrenocorticotropic hormone (ACTH, 31 [2.7%]). Focal seizures were controlled by treatment in 672 (58.7%) patients, while not controlled in 411 (35.9%) patients. The next most common seizure type reported was infantile spasms (679 [38.8%]). Of the 660 patients who received treatment for infantile spasms, 517 (78.3%) were treated with GABAergics and 118 (17.9%) with ACTH, either as monotherapy or in combination with other therapies. Infantile spasms were controlled by treatment in 471 (71.5%) patients, while not controlled in 105 (15.9%) patients.

### TSC-associated neuropsychiatric disorders (TAND)

Among the patients who were evaluated for TAND, academic/scholastic difficulties were noted in 682 (57.8%) patients. Of the 822 (39.2%) patients who had been evaluated using intelligent quotient (IQ)-type tests, 451 (54.9%) patients had mild to profound intellectual disability. Autism spectrum disorder (ASD), attention deficit hyperactivity disorder (ADHD), anxiety disorder, and depressive disorder were reported in 20.7, 19.6, 9.1, and 6.1% of patients excluding patients whose data was not available. The mean (median [range]) age at diagnosis of neuropsychiatric disorders were — ASD, 7.6 (5 [0–38]); ADHD, 7.7 (6 [0–38]); anxiety, 17.8 (15 [0–50]); depression, 24.4 (21 [3–49]) years (Additional file 1). Neuropsychological skills were assessed in 510 patients of whom 281 (55%) patients had performance < 5^th^ percentile. Patients who reported at least one behavioral problem were 745 (35.6%). Behavioral problems reported in > 10% patients were sleep difficulties, impulsivity-overactivity, severe aggression, anxiety, and mood swings. Missing data of TAND features in the TOSCA population was high (Table 3).

**Table 3:** TSC-Associated Neuropsychiatric Disorders (TAND) features reported in TOSCA at baseline

TAND features	Patients with manifestation, n/patients with available data (%)	Patients with available data, n/2093 (%)	Missing data, n/2093 (%)
Behavioral difficulties			
Overactivity	317 (20.7)	1533 (73.2)	560 (26.8)
Sleep difficulties	301 (19.5)	1540 (73.6)	553 (26.4)
Impulsivity	297 (19.4)	1533 (73.2)	560 (26.8)
Anxiety	205 (13.7)	1498 (71.6)	595 (28.4)
Mood swings	201 (13.4)	1499 (71.6)	594 (28.4)
Severe aggression	168 (10.8)	1559 (74.5)	534 (25.5)
Depression mood	124 (8.3)	1500 (71.7)	593 (28.3)
Self-injury	106 (6.8)	1555 (74.3)	538 (25.7)
Obsession	92 (6.1)	1505 (71.9)	588 (28.1)
Psychosis	34 (2.3)	1514 (72.3)	579 (27.7)
Hallucination	23 (1.5)	1512 (72.2)	581 (27.8)
Psychiatric disorders			
ASD	291 (20.7)	1406 (67.1)	687 (32.8)
ADHD	260 (19.6)	1329 (63.4)	764 (36.5)
Anxiety	118 (9.1)	1294 (61.8)	799 (38.2)
Depression	80 (6.1)	1301 (62.1)	792 (37.8)
Intellectual Ability^a^			
Normal	371 (45.1)	NA	NA
Mild ID	232 (28.2)	NA	NA
Moderate ID	123 (15.0)	NA	NA
Severe ID	75 (9.1)	NA	NA
Profound ID	21 (2.6)	NA	NA
Academic difficulties			
Patients ever had difficulties in academic performance	682 (57.8)	1179 (56.3)	914 (43.7)
Patients assessed for academic difficulties	332 (48.6% of those with reported difficulties)	332 (15.9)	NA
Neuropsychological difficulties			
Patients ever had any neuropsychological skill assessed	510 (40.1)	1270 (60.6)	823 (39.3)
Patients with performance < 5^th^ percentile	281 (55% of those who had neuropsychological assessment)	281 (13.4)	NA

*ASD* autism spectrum disorder, *ADHD* attention deficit hyperactivity disorder, *NA* not applicable, *TOSCA* TuberOus SClerosis registry to increase disease Awareness, *TAND* TSC-associated neuropsychiatric disorders, *ID* Intellectual Disability

^a^Patients with intellectual ability measured by intellectual quotient were available in 822 (39.3%) out of 834 patients. Intelligent ability was not measured in 752 (35.9%) patients

### Renal manifestations

Renal angiomyolipomas were reported in 987 (47.2%; males, 42.5% and females, 57.5%) patients and diagnosed at a mean age of 17.4 years (median age 13.0 years; range 0–67) (Table 2). The majority of angiomyolipomas (946 patients, 95.8%) recorded at baseline were diagnosed prior to recruitment, most (792 patients, 83.7%) of which were without any signs and symptoms at the time of assessment. Of the patients with ongoing lesions, 793 (83.8%) had bilateral angiomyolipomas, 829 (87.6%) had multiple lesions, 329 (34.8%) had angiomyolipoma lesions > 3 cm in diameter, and 396 (41.9%) had both multiple and bilateral renal angiomyolipomas. The most common past medical history of signs/symptoms reported included pain (51 [5.4%]), elevated blood pressure (48 [5.1%]), impaired renal function (36 [3.8%]), hemorrhage (47 [5%]), and microscopic hematuria (35 [3.7%]). Renal angiomyolipomas were treated in 274 (27.8%) patients. Most common mode of treatment (monotherapy or in combination with other treatment modalities) was embolization (126 [46%]) followed by mTOR inhibitors (110 [40.1%]).

Among other renal features, multiple renal cysts were the most frequent (22.8%) while polycystic kidneys (3.5%), impaired renal function (non-angiomyolipoma related; 2.1%), and renal malignancy (1.1%) were infrequently reported (Table 2).

### Pulmonary manifestations

Lymphangioleiomyomatosis (LAM) was reported in 144 (6.9%) patients of whom 142 (98.6%) were adults >18 years (Table 2). Of these, 136 were females (≤18 years, 2 [1.4%]; 18–40 years, 70 [51.4%]; > 40 years, 64 [47%]) and 8 were males. Mean age at diagnosis was 36.7 years (median age 35.0 years; range 9–61). Almost all patients (142 of 144) were diagnosed with LAM when they were > 18 years of age. LAM caused signs and/or symptoms in 58 (40.3%) patients. The most common symptom was dyspnea (69%), and lung collapse and/or pneumothorax (44.8%). In the 46 patients who received treatment, mTOR inhibitors (23 patients, 50%, alone or in combination with other treatment modalities) was most commonly used. Other treatment modalities included surgery, chest tube, chylous fluid drainage, and bronchodilators.

### Cardiovascular manifestations

Cardiac rhabdomyomas, which were the most frequent cardiovascular manifestations found in 717 (34.3%) patients (Table 2). These were diagnosed at a mean age of 3.1 years. Cardiac rhabdomyomas reported earlier and were still present in 483 (67.4%) patients at the time of assessment, resolved spontaneously in 208 (29%) patients, and resolved after treatment in 24 (3.3%) patients. Among other cardiovascular features, arrhythmia/dysrhythmia and valve dysfunction were reported in 5.6 and 2.9% of patients, respectively. Aneurysm (1%) and coarctation of aorta (0.2%) were rarely reported.

### Dermatological and dental manifestations

The most frequently reported dermatological manifestations were facial angiofibromas (1199 [57.3%]). The median age at onset of facial angiofibroma was 6.0 years (range 0–67). Approximately, one-third (32.8%) of the patients had received treatment. Common treatment modalities included laser therapy (49.1%), topical mTOR inhibitors (23.2%) and systemic mTOR inhibitors (21.1%) used alone or in combination with other treatment modalities. Hypomelanotic macules (≥3) were reported in 66.8% of patients (median age 1.0 year; range, 0–67). Other dermatological manifestations included shagreen patch (27.4%), ungual or peri-ungual fibroma (16.7%), forehead plaque (14.1%), confetti lesions (8.6%), and other dermatological conditions (17.2%, angiomyolipoma [also known as a cutaneous angiolipoleiomyoma], cafe au lait macule, poliosis, and skin tags). Dental manifestations included randomly distributed pits in dental enamel (98 [4.7%]) and gingival fibromas (96 [4.6%]).

### Ophthalmological manifestations

Retinal hamartomas, the most frequent ophthalmological manifestation, were reported in 294 (14%) patients, and diagnosed at a mean age of 8.3 years (median age 5.0 years; range 0–50). These were symptomatic in 12.6% of patients. Symptoms included blurred vision, constriction of visual field, and visual impairment. Retinal achromic patch (53 [2.5%]) and other TSC-related ophthalmological lesions (73 [3.5%]) were also reported but less commonly.

### Other manifestations

Liver hamartomas were reported in 190 (9.1%) patients, with a higher frequency in female patients (73.7% of those with liver hamartomas), and diagnosed at a mean age of 23.3 years (median age 22 years; range 0–61). Both ongoing liver hamartoma and angiomyolipomas was reported in 168 patients.

Reproductive abnormalities were noted in a small number of patients and included menstrual cycle disorders (67 [6.2%]), amenorrhea (female patients > 11 years, 38 [3.5%]), abnormal onset of puberty (93 [4.4%]), other abnormal reproductive conditions (49 [2.3%]), abnormal hormone levels including prolactin (21 [1%]), thyroid-stimulating hormone (145 [6.9%]), follicle-stimulating hormone (37 [1.8%]), testosterone (21 [1%]), and luteinizing hormone (35 [1.7%]).

Collectively, manifestations previously thought to be rare were reported in 316 (15.1%) patients; bone sclerotic foci in 87 patients, scoliosis in 46 patients, thyroid adenoma in 15 patients, spleen angiomyolipoma in 5 patients, pancreatic neuroendocrine tumor in 5 patients, and hemihypertrophy (abnormal growth on one side of the body), calvarial sclerosis and thickening (each in 2 patients).

Co-morbidities were reported in 347 (16.6%) patients; cardiovascular co-morbidities were the most frequent (44 patients). Other less common comorbidities included malignancies (15 patients), dyslipidemia (17 patients) and diabetes (5 patients). Other features of TSC reported were bone cysts (65 patients), non-renal hamartoma (excluding liver, 34 patients), and hamartomatous rectal polyps (8 patients).

### Manifestations of TSC across age groups in TOSCA Participants

**Fig. 4:**
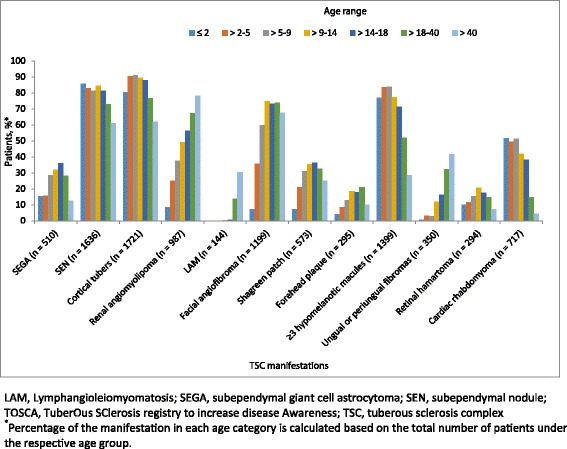
Major manifestations of TSC categorized by age range in TOSCA participants (*N* = 2093). LAM, Lymphangioleiomyomatosis; SEGA, subependymal giant cell astrocytoma; SEN, subependymal nodule; TOSCA, TuberOus SClerosis registry to increase disease Awareness; TSC, tuberous sclerosis complex. *Percentage of the manifestation in each age category is calculated based on the total number of patients under the respective age group

Figure 4 depicts a distinctive pattern to age-related emergence and prevalence of TSC manifestations. Hypomelanotic macules, subependymal nodules, cortical tubers and cardiac rhabdomyomas were reported from age ≤ 2, (presumably as soon as they were looked for), and their prevalence did not change. In contrast, the prevalence of SEGAs and retinal hamartomas peaked in childhood, and the prevalence of renal angiomyolipomas, facial angiofibromas, forehead plaques and shagreen patches went on increasing into adulthood. However all these lesions were reported in a small number of patients from age ≤ 2 years. In contrast, pulmonary LAM and ungual fibromas presented later but also became more prevalent in adults.

## Discussion

The TOSCA natural history study represents the largest clinical collection of TSC data to date. It is not possible to derive an accurate absolute prevalence of TSC or its individual manifestations in the general population from this dataset because it was ascertained from a specialist clinic population. However, the data do record the relative prevalence of different manifestations within this cohort and their natural history. Reassuringly, our data shows a number of similarities to what has been reported in previous studies of TSC [1, 14]. For instance, *TSC2* mutations were more common than *TSC1* mutations [4, 15] and the prevalence of certain disease features such as cortical tubers, subependymal nodules and epilepsy were similar to previous reports [1, 14]. However, the frequencies of some of the other features such as SEGA, TAND, and renal angiomyolipomas differed from the published data [1, 14]. Potential reasons for these similarities and differences are discussed in more details below.

SEGAs were reported in 24.4% of the patients, which is much higher than the previously reported rate of 10–15% [1, 11]. The higher frequency of SEGAs seen in this cohort could be because the majority of centers included in TOSCA were specialist neurology centers. There also is ongoing discussion with respect to the most accurate definition of SEGA, which may have been of influence on the number of SEGA reported in TOSCA. In 2012, a European Consensus Group defined SEGA as a tumor in a TSC patient that is usually characterized by a location near the foramen of Monro, > 0.5 cm in diameter, with any documented growth, and gadolinium enhancement on neuroimaging [16]. Later that year, an international panel of experts defined SEGA as a lesion at the caudothalamic groove with either a size of more than 1 cm in any direction or a subependymal lesion at any location that has shown serial growth on consecutive imaging regardless of size [17]. Most SEGAs show avid enhancement after contrast administration; however, a growing subependymal lesion even in the absence of enhancement should be considered a SEGA [17]. Median age at SEGA diagnosis was 8 years but more than a quarter of patients were diagnosed with SEGA already before age two years, highlighting the young age at onset and early need for follow-up. When compared to the last scan, 36.7% of ongoing SEGAs were reported to have grown in size. Since SEGAs are known to grow over time, there are existing recommendations for their regular follow-up and timely management [10, 16]. Median time between scans was 1 year and median time between SEGA diagnosis and start of treatment was less than 1 year, reflecting good clinical practice with respect to SEGA follow-up and management in the TOSCA cohort. Although SEGA growth was most common between ages 5–18 years, growth after age 18 years remains possible as was shown in this cohort. This highlights the necessity to stay vigilant to possible symptoms related to SEGA-growth also at adult age. In this analysis, surgery was the most common mode of treatment for SEGAs followed by mTOR inhibitors. Until the recent approval of everolimus for the treatment of SEGAs associated with TSC [9, 18], surgery was the only treatment option. For acutely symptomatic SEGAs, surgery and cerebrospinal fluid diversion remain the treatment of choice [10]. However, for asymptomatic growing SEGAs, mTOR inhibitors may be considered especially in multisystem disease [10], since mTOR inhibitors have also been found to benefit other manifestations of TSC [19–23]. Due to high rate of regrowth of residual tumors [24], mTOR inhibitors should be recommended for those patients with SEGA, in whom total surgical removal of SEGA is not possible. Complete SEGA resection might be more difficult to achieve in the presence of bilateral SEGA [17], which were present in over one third of patients in this cohort.

Similar to previous reports [3], this analysis of TOSCA data showed that epilepsy (83.5%) was the most commonly reported clinical presentation of TSC. Focal seizures were the most common type of seizures followed by infantile spasms. GABAergics (vigabatrin) were most frequently used, both for focal seizures and for infantile spasms. This finding is in line with European recommendations made by TSC Consensus meeting for SEGA and epilepsy management in 2012, which recommended vigabatrin both for infantile spasms and focal seizures in infants in the first year of life [25]. Also the guidelines from the 2012 International TSC consensus Conference recommend vigabatrin as first line and adrenocorticotropic hormone as the second line treatment for infantile spasms in individuals with TSC [10]. The use of other anti-epileptic drugs; e.g., sodium channel blockers and fructose derivatives will be described in more detail in a subsequent paper. Despite a high rate of refractory epilepsy, alternative treatment options such as the ketogenic diet and epilepsy surgery were not commonly used in this population.

Recently, mTOR inhibitors have been thought to be useful for the treatment of refractory seizures [26–28]. Results from a phase III, randomized, double-blind, placebo-controlled study (EXIST-3; NCT01713946) could tell us the efficacy and safety of 2 trough-ranges of everolimus (an mTOR inhibitor) as adjunctive therapy in patients with TSC who have refractory seizures.

The TAND domain showed lower rates of behavioral and psychiatric disorders than previously reported [29–31]. The rates of intellectual disability were similar to previous reports [29, 30] but there was no clear evidence of a bimodal distribution of IQ/DQ. Very high rates of academic difficulties and of neuropsychological deficits were reported, and represent the first report of the potential magnitude of academic and neuropsychological deficits in TSC. Strikingly though, diagnoses of ASD, ADHD, anxiety and depression were made very late, and the TAND domain was characterized by very high rates of missing data. These findings suggest that, even in the TOSCA cohort, TAND is underdiagnosed and therefore undertreated. A major challenge in maintaining a database like TOSCA is to ensure that data are complete, especially since these are collected from many centers over a long time period. Missing data for TAND suggests that a considerable number of patients were never assessed for TAND. It is apparent that there is a specific need to educate clinicians to assess all the patients with TSC for TAND. In order to address the need, to increase the awareness of TAND and the importance of screening for these difficulties, the Neuropsychiatry Panel at the 2012 Tuberous Sclerosis Complex International Consensus Conference developed a simple tool called the TAND checklist [31, 32]. The neuropsychiatry panel recommended at least an annual screening for TAND and comprehensive formal evaluation for TAND at key developmental time points: infancy (0–3 years), preschool (3–6 years), pre-middle school (6–9 years), adolescence (12–16 years), early adulthood (18–25 years), and as needed thereafter. Management strategies should be based on the TAND profile of each patient and should be based on evidence-based good practice guidelines/practice parameters for individual disorders (e.g., autism spectrum disorder, attention deficit hyperactivity disorder, anxiety disorder). The TAND checklist can serve as an ideal guide to facilitate the discussion between the healthcare professionals and patients [31].

The lower rate of renal angiomyolipomas reported in this population was likely attributable to the fact that the cohort had a younger median age. This is clear from Fig. 4 that the prevalence in adults (e.g., 80% in age > 40) is the same as in other studies. Also, the lower than expected prevalence of complications of angiomyolipoma such as hypertension, microscopic hematuria, and impaired renal function (reported in the literature as 27, 25–50, and 40% respectively) [33–35] reported here is a reflection of the young age of this cohort, who have not had time to develop the most common adult renal complications; and to good practice in specialist clinics of active surveillance and pre-emptive treatment of enlarging angiomyolipomas. The finding that 27.8% of those with angiomyolipomas had received treatment for them (presumably mostly pre-emptive) supports this suggestion. An important finding is that a significant number of children (55 patients aged <18 years) needed intervention for their angiomyolipomas. It is also of note that the occurrence of angiomyolipomas was not statistically different in women compared to men. A previous report [36] found angiomyolipomas complications were more common in women than men, and two thirds of the recruits for EXIST-2 [8] were women; both findings implying that if there is no sex difference in angiomyolipomas prevalence, women are more vulnerable to developing complications. Renal angiomyolipomas can cause considerable morbidity including complications like aneurysm and hemorrhage [37]. Moreover, renal complications have been associated with the most common cause for death in adult patients with TSC [38]. Timely diagnosis and treatment are therefore of utmost importance. The main objective in treating renal angiomyolipomas is to prevent hemorrhage and to preserve renal function. TSC Consensus Conference guidelines recommend embolization followed by corticosteroids as the first-line of treatment for angiomyolipomas presenting with acute hemorrhage [10]. An mTOR inhibitor is the recommended first-line therapy for asymptomatic, growing angiomyolipoma > 3 cm in diameter [10]. In this cohort, renal angiomyolipomas were most commonly treated with embolization followed by mTOR inhibitors.

LAM is the main pulmonary manifestation of TSC, which is seen in about 40% of women of reproductive age [39, 40]. A recent study has reported a higher prevalence (up to 80%), especially in women over 40 years of age [39]. In this dataset, LAM was reported much less frequently compared to what has been published in literature. This could probably be explained partly by the young average age of the cohort and probably also because screening may have been based on clinical symptoms rather than high-resolution chest computed tomography (HRCT). However 40% of these patients were symptomatic from their LAM. Cudzilo et al. reported that most women with TSC develop cystic changes consistent with LAM [39], highlighting the importance of routine surveillance using CT scan. As per the guidelines, baseline pulmonary function testing and HRCT must be performed in females 18 years or older, even if asymptomatic [10]. Screening for LAM in female patients and adult males (symptomatic) with TSC as per the recommendations would be helpful. The high occurrence of symptomatic retinal hamartomas (12.6%) is a new finding and has implications for surveillance. In this analysis of TOSCA, it was also noted that most of the patients were asymptomatic. It is therefore crucial to highlight the importance of regular surveillance in all patients with TSC even in the absence of symptoms to help prevent complications.

With regard to the age at occurrence, most of the manifestations in majority of the patients were diagnosed at the median age of 1 year. The mean age at diagnosis for cardiac rhabdomyoma was 3.1 years. The highest incidence of cardiac tumors is in children below 2 years of age reaching up to 65–80% [40]. This late age at diagnosis of cardiac rhabdomyomas could be related to age at diagnosis of TSC. Apart from those diagnosed with rhabdomyomas on fetal ultrasonography, other patients may have had an echocardiogram organized after their diagnosis of TSC was made.

Early, sometimes prenatal, diagnosis of TSC enables the beginning of surveillance and thus prevention or amelioration of complications such as epilepsy, intellectual disability, autistic behaviors and tumors development [17, 41, 42]. As TSC is a genetic disease, the family members must also be assessed. Family counselling must be done and genetic testing carried out [10]. Current molecular techniques enable the *TSC1/TSC2* mutation detection in more than 95% of patients [4]. In TOSCA participants, genetic testing was not reported in about 40% of the patients, which might be due to ethical or financial reasons. Five patients reported both *TSC1* and *TSC2* mutations, this unexpected finding is being investigated and will be reported in more detail in future publications.

Though TOSCA is a large clinical case series, it must be noted that patients were recruited through clinical centers with expertise in TSC and milder cases may not always be seen at these centers. The study design therefore includes potential limitations inherent to clinical case series, albeit large-scale, multinational ones. Nevertheless, participation of a large number of centers with complementary expertise has helped inclusion of a huge number of patients with TSC, which reveals occurrence rates of complications that are likely to be representative of hospital clinical practice. This baseline paper of TOSCA provides a detailed description of the disease trajectory of TSC. The registry can provide valuable insights into the necessity for monitoring, the timing, and the indications for treatment of this disease. Further follow-up studies of TOSCA including research projects will provide more detail in understanding the treatment interventions and outcomes.

## Conclusion

This international registry provides a better understanding of the TSC manifestations, and facilitates development of better management and surveillance strategies for patients with TSC. Following patients over the years will help in understanding any changes in the treatment and outcome of the different manifestations. Baseline analysis has highlighted the serious import of epilepsy and SEGA in children, and renal angiomyolipomas in children as well as adults. We found inadequate surveillance for TAND; hopefully this will be improved in future by widespread adoption of the international guidelines including use of the TAND checklist. Clinicians need to be alert for rare complications but especially changes in retinal hamartomas. We believe comprehensive surveillance will lead to more pro-active pre-emptive treatment and better outcomes in future. Subsequent analyses are planned yearly to allow the clinical course of the disease over time to be evaluated.

## Additional file

Additional file 1: TableS1. Age and Gender Distribution of TSC manifestations reported in TOSCA. (DOCX 13 kb)

## Abbreviations

ACTH: Adrenocorticotropic hormone
ADHD: Attention deficit hyperactivity disorder
ASD: Autism spectrum disorder
GABA: Gamma-aminobutyric acid
HRCT: High-resolution chest computed tomography
LAM: Lymphangioleiomyomatosis
mTOR: Mammalian target of rapamycin
SEGA: Subependymal giant cell astrocytoma
TAND: TSC-associated neuropsychiatric disorders
TOSCA: **T**uber**O**us **SC**lerosis registry to increase disease **A**wareness
TSC: Tuberous sclerosis complex
