# Supplementary material for: TuberOus SClerosis registry to increase disease Awareness (TOSCA) – baseline data on 2093 patients
Source: Orphanet J Rare Dis. 2017 Jan 5;12:2. doi: 10.1186/s13023-016-0553-5 (PMC5217262; doi:10.1186/s13023-016-0553-5)
Supplement: Additional file 1: TableS1. — Age and Gender Distribution of TSC manifestations reported in TOSCA. (DOCX 13 kb) [file 13023_2016_553_MOESM1_ESM.docx]

**Supplementary Table: Age and Gender Distribution of TSC manifestations reported in TOSCA**

| **Manifestation**  **Characteristics** | **Baseline data** |
| --- | --- |
| Subependymal giant cell astrocytoma, n (%) | 510 (24.4) |
| Age at diagnosis, years  Mean (SD)  Median (range) | 10.8 (10.8)  8 (0-51) |
| Gender, n (%)  Male  Female | 257 (50.4)  253 (49.6) |
| Renal angiomyolipoma, n (%) | 987 (47.2) |
| Age at diagnosis, years  Mean (SD)  Median (range) | 17.4 (14.6)  13 (0-61) |
| Gender, n (%)  Male  Female | 419 (42.5)  568 (57.5) |
| Lymphangioleiomyomatosis, n (%) | 144 (6.9) |
| Age at diagnosis, years  Mean (SD)  Median (range) | 36.7 (11.4)  35 (9-61) |
| Gender, n (%)  Male  Female | 8 (5.6)  136 (94.4) |
| Autism spectrum disorder, n (%) | 291 (13.9) |
| Age at diagnosis, years  Mean (SD)  Median (range) | 7.6 (7.03)  5 (0-38) |
| Gender, n (%)  Male  Female | 186 (63.9)  105 (36.1) |
| Attention deficit hyperactivity disorder, n (%) | 260 (12.4) |
| Age at diagnosis, years  Mean (SD)  Median (range) | 7.7 (6.46)  6 (0-38) |
| Gender, n (%)  Male  Female | 161 (61.9)  99 (38.1) |
| Depressive disorder, n (%) | 80 (3.8) |
| Age at diagnosis, years  Mean (SD)  Median (range) | 24.4 (12.24)  21 (3-49) |
| Gender, n (%)  Male  Female | 28 (35)  52 (65) |
| Anxiety disorder, n (%) | 118 (5.6) |
| Age at diagnosis, years  Mean (SD)  Median (range) | 17.8 (12.95)  15 (0-50) |
| Gender, n (%)  Male  Female | 50 (42.4)  68 (57.6) |
